# Preoperative brain injury and neurodevelopmental outcomes in syndromic and non-syndromic children with complex congenital heart disease: a single-centre cohort study

**DOI:** 10.1007/s00431-026-07437-w

**Published:** 2026-09-29

**Authors:** Chiara Paolin, Alice Pozza, Claudio Ancona, Laura Mezzalira, Paola Cogo, Giovanni Di Salvo, Massimo Padalino, Fabio Scattolin, Vladimiro Vida, Stefano Sartori, Renzo Manara

**Affiliations:** 1 Paediatric Neurology and Neurophysiology Unit, Department of Women’s and Children’s Health, University of Padova, Padua, Italy; 2 Paediatric Cardiology Unit, Department of Women’s and Children’s Health, University of Padova, Padua, Italy; 3 School of Medicine, University of Ferrara, Ferrara, Italy; 4 Department of Medical Area, DAME - Division of Paediatrics, University of Udine, Udine, Italy; 5 Paediatric and Congenital Cardiovascular Surgery, Department of Precision and Regenerative Medicine and Ionian Area, University of Bari “Aldo Moro”, Bari, Italy; 6 Paediatric and Congenital Cardiac Surgery Unit, Department of Cardiac, Thoracic and Vascular Sciences, University of Padova, Padua, Italy; 7 Neuroradiology, Department of Neurosciences, University of Padova, Padua, Italy

**Keywords:** Congenital heart disease, Syndromic children, Brain magnetic resonance, Neurodevelopment

## Abstract

Children with complex congenital heart disease (CHD) are at high risk of brain lesions both before and after cardiac surgery. Preoperative brain injury reflects foetal and perinatal brain development and represents an independent predictor of neurodevelopmental outcome. This study aimed to characterize preoperative brain lesions and their correlation with clinical course and neurodevelopmental outcomes in children with complex CHD. The secondary objective was to compare findings between syndromic and non-syndromic patients, as preoperative brain injury in syndromic children remains underreported. A single-centre cohort study was performed at the University of Padova, Italy, involving children with complex CHD who underwent preoperative brain MRI between January 2020 and October 2024. Patients were categorized based on the presence or absence of a known or suspected genetic syndrome. Neurological and neurodevelopmental evaluations were conducted pre- and postoperatively, and at 6- and 18-months follow-up. Among 54 enrolled patients, 21 (39%) had a suspected or confirmed genetic syndrome. Preoperative brain lesions were present in 24 children (44%), including 16/33 (48%) non-syndromic and 8/21 (38%) syndromic patients (OR 0.65 [0.21–1.94], *p* = 0.5708). Arterial ischemic stroke was the most common lesion (20%), followed by subdural haemorrhage (11%), cerebral sinovenous thrombosis (9%), and white matter injury (6%). Despite similar lesion rates, syndromic children showed more complicated perinatal histories and greater neurodevelopmental impairment, particularly in motor and language domains.

*Conclusion*: Syndromic children with CHD do not have higher rates of preoperative brain injury but remain at greater neurodevelopmental risk. Further studies are warranted to confirm these findings.

**Table:** 

**What is Known:** • *Children with complex congenital heart disease (CHD) are at high risk for preoperative brain injury and neurodevelopmental impairment.* • *Syndromic children with CHD are often excluded from neurodevelopmental studies.*	
**What is New:** • *Preoperative brain lesions (44%), mainly arterial ischemic stroke (20%), are frequent in our cohort of syndromic and non-syndromic children with complex CHD.* • *Syndromic children with CHD do not show increased rates of preoperative brain injury compared to non-syndromic children, but remain a population at higher neurodevelopmental risk.*

**Supplementary Information:**

The online version contains supplementary material available at https://doi.org/10.1007/s00431-026-07437-w.

## Introduction

Congenital heart disease (CHD) is the most common birth defect, affecting 1 in every 8000 children. Over the past years, advances in diagnostic tools and cardiac surgery techniques have enabled more than 97% of children born with CHD to reach adulthood. Besides survival, much interest has been conveyed to functional concerns, as adverse neurodevelopmental outcomes are increasingly recognized in children affected with CHD [1–4]. Overall, CHD encompasses a broad spectrum of diseases, from simple lesions to more complex defects, the latter requiring open heart surgery within the first year of life and presenting an increased risk of adverse neurodevelopmental outcomes [5–7].

Across classification of heart defects, isolated or non-syndromic CHD defines lesions in which the heart is the only affected structure, whereas syndromic CHD refers to the presence of additional developmental defects [8].

Recent literature has been focusing on the incidence and significance of brain lesions in children with complex CHD, both pre- and postoperatively.

Preoperative brain lesions reflect foetal/perinatal brain growth and susceptibility to damage, and represent an independent risk factor for neurodevelopmental outcome [9]. White matter injury (WMI), Arterial ischemic stroke (AIS) and microhaemorrhages are the most common lesions in preoperative scans and their presence has been largely described [8, 10–13]. However, syndromic children have been excluded from most of the literature assessing neurodevelopmental outcomes in complex CHD.

The primary aim of the current study was to characterize preoperative brain lesions in a cohort of syndromic and non-syndromic children with complex CHD, and to examine their correlation with clinical course and neurodevelopmental outcome. A secondary objective was to compare neurodevelopmental outcomes between syndromic and non-syndromic children.

## Material and methods

### Study design

This single-centre cohort study was performed at the Department of Women’s and Children’s Health, University of Padova, Italy, between January 2020 and October 2024.

### Study population

Patients were considered eligible for the study if they were diagnosed with complex CHD requiring cardiac surgery within the first year of life and underwent a brain MRI study before cardiac surgery. Exclusion criteria were severe hemodynamic instability or severe hepatic or renal insufficiency requiring intensive care before surgery. Patients were subdivided according to presence or absence of known/suspected genetic syndrome (i.e. Di George or Down syndrome). A genetic syndrome was considered suspected in the presence of multiple dysmorphic features in patients with inconclusive or ongoing genetic testing.

Patient characteristics as well as surgical and medical data were extracted from the electronic clinical records of our institution and collected in a Research Electronic Data Capture database. Clinical data extracted from medical records included: prenatal (maternal conditions, prenatal diagnosis), perinatal (type of delivery, APGAR score), demographic (sex, anthropometrics, CHD type), presurgical (time to surgery/MRI, preoperative interventions), surgical (cardiopulmonary bypass (CPB) details) and neurological (neurological and neurodevelopmental evaluations) factors.

All children underwent preoperative brain MRI on the same 1.5 T scanner (Philips Achieva, Best, the Netherlands). MRI protocol included T1, T2, diffusion and T2* weighted imaging, phase contrast MR-venography and Time-of Flight (TOF) Magnetic Resonance Angiography.

Lesion type, number and location were recorded on preoperative brain MRIs according to the European Association Brain in Congenital Heart Disease Consortium [12]. Postoperative brain MRI was performed whenever clinically required and included scans from the above-mentioned protocol.

Clinical, cardiological and neurodevelopmental evaluations were performed before and after surgery and afterwards at 6 and 18 months of age. Cardiological evaluation consisted of comprehensive structural echocardiography, performed by two paediatric cardiologists (A.P., G.DS.) using Vivid E95ultrasound system (General Electric Healthcare, Horten, Norway). Neurodevelopmental evaluations were performed by two paediatric neurologists (C.P. and S.S.) using Hammersmith Neonatal and Infant Neurologic Evaluation and Griffiths III (2nd edition) scales as a reference.

The study was approved by the local hospital Research Board, University of Padova (Protocol ID 3142/AO/14), and parental written informed consent was obtained for included patients. All data were collected while maintaining confidentiality and were anonymized for statistical analysis.

### Statistical analysis

The continuous variables are displayed as mean ± standard deviation, if normally distributed, or as median and interquartile range (IQR), if not normally distributed. The normality distribution was tested using the Shapiro–Wilk test. The binomial and ordinal qualitative variables are presented as frequencies and percentages. In univariate analyses, chi-square test of proportions for categorical variables was used to assess differences. When expected frequencies were low, Fisher’s exact test was preferred. Univariate analyses were not adjusted for multiple comparisons.

To evaluate the mechanism of missing data across variables, Little’s Missing Completely at Random (MCAR) test was performed. A *p*-value > 0.05 was considered indicative of data missing completely at random (MCAR). Complete-case analyses were applied for subgroup comparisons.

To account for baseline imbalances between groups (such as intrauterine growth retardation (IUGR), birth weight, and delivery mode), adjusted comparisons for primary neurodevelopmental outcomes at follow-up (language and motor delay) were performed using stratified Mantel–Haenszel tests, reporting common Odds Ratios (OR) and 95% confidence intervals (95% CI).

All statistical analyses were performed with SPSS software version 25.0 (International Business Machines Corporation, Armonk, NY, USA).

## Results

### Demographics

**Fig.1:**
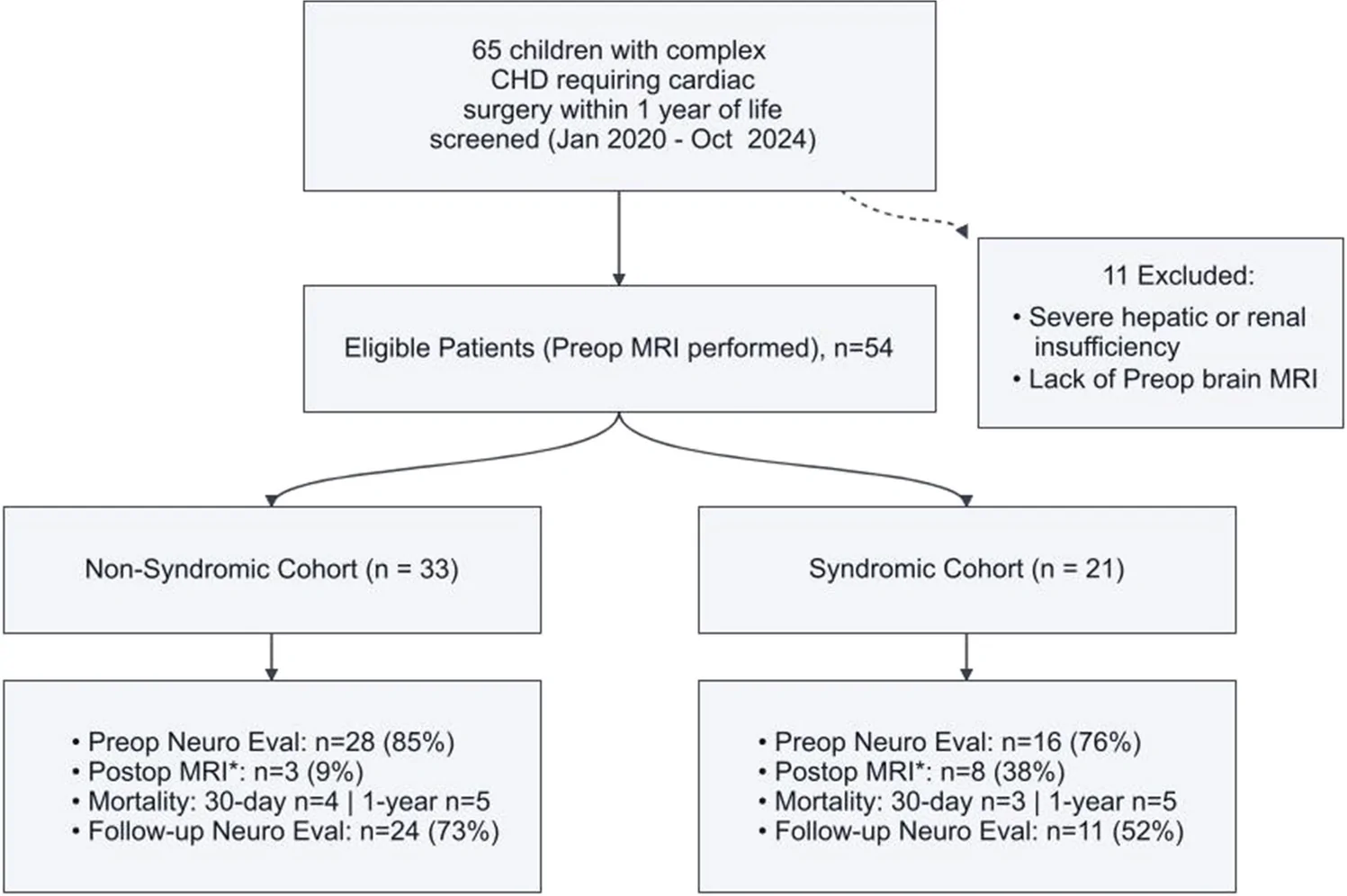
STROBE Patient Flow Diagram. Flowchart illustrating patient screening, eligibility, subgroup allocation (non-syndromic vs. syndromic), availability of postoperative brain MRI scans, preoperative and follow-up neurological assessments and mortality. *Postoperative MRI performed for patient related clinical factors. (CHD: congenital heart disease; Eval: evaluation)

Overall, 54 consecutive eligible children (34 males) were enrolled, and 21/54 (39%; 12 males) had a known or suspected genetic syndrome. Figure 1 illustrates the STROBE Patient Flow Diagram. Clinical characteristics of all patients are summarized in Table 1. Maternal hypertension, gestational diabetes and hypo/hyperthyroidism were recognized in 11/54 (20%). Seven children (13%) were born of twin pregnancy, four of them were syndromic and 3 non-syndromic (*p* = 0.29). IUGR was more common among syndromic children (5/21 vs 1/33; OR 10.00 [1.08–92.83], *p* = 0.03). Prenatal diagnosis of complex CHD was available in 37/54 (69%). Caesarean section was more common among syndromic children (14/21 vs 8/33; OR 6.25 [1.83–21.32], *p* = 0.004), while emergent caesarean section did not reach a statistically significant difference among the two subgroups. Syndromic children had significantly lower birth weights compared to non-syndromic peers (MD = −250 g [−640 to + 140], *p* = 0.016). Cardiac surgery was performed at a median age of 31 (11–126) days. Preoperative cyanosis was observed in 20 (37%) children. Cardiac surgery with CPB was performed in 47 (87%). Mortality at 30 days and 1 year after surgery did not differ between non-syndromic and syndromic children (4/33 vs 3/21, *p* = 1, and 5/33 vs 5/21, *p* = 0.48, respectively).

**Table 1:** Clinical characteristics of the study population

	All Patients (*N* = 54)	Non-Syndromic (*N* = 33)	Syndromic (*N* = 21)	Odds Ratio [95% CI]	*p*-value
Male sex	34 (63.0%) [49.6–74.7]	22 (66.7%) [49.7–80.4]	12 (57.1%) [36.5–75.5]	0.67 [0.22–2.07]	0.468
Maternal conditions*	11 (20.4%) [11.8–32.9]	7 (21.2%) [10.7–37.8]	4 (19.0%) [7.7–38.6]	0.87 [0.22–3.41]	0.848
Twin pregnancy	7 (13.0%) [6.4–24.3]	3 (9.1%) [3.1–23.6]	4 (19.0%) [7.7–38.6]	2.35 [0.47–11.75]	0.288
IUGR	6 (11.1%) [5.2–22.2]	1 (3.0%) [0.5–15.3]	5 (23.8%) [10.6–45.1]	10.00 [1.08–92.83]	0.028
Prenatal diagnosis	37 (68.5%) [55.3–79.1]	23 (69.7%) [52.7–82.6]	14 (66.7%) [45.4–82.8]	0.87 [0.27–2.82]	0.816
Cesarean section	22 (40.7%) [28.7–54.0]	8 (24.2%) [12.4–41.9]	14 (66.7%) [45.4–82.8]	6.25 [1.83–21.32]	0.004
Emergent C section	8 (14.8%) [7.7–26.4]	3 (9.1%) [3.1–23.6]	5 (23.8%) [10.6–45.1]	3.12 [0.66–14.73]	0.238
Vaginal induced delivery	9 (16.7%) [9.0–28.7]	7 (21.2%) [10.7–37.8]	2 (9.5%) [2.6–28.1]	0.39 [0.07–2.12]	0.456
Term (≥ 39 wk)	22 (40.7%) [28.7–54.0]	13 (39.4%) [24.7–56.3]	9 (42.9%) [24.5–63.5]	1.15 [0.38–3.53]	0.801
Early term (37–39 wk)	17 (31.5%) [20.6–44.8]	12 (36.4%) [22.2–53.4]	5 (23.8%) [10.6–45.1]	0.55 [0.16–1.88]	0.334
Late preterm (34–37 wk)	7 (13.0%) [6.4–24.3]	2 (6.1%) [1.7–19.6]	5 (23.8%) [10.6–45.1]	4.84 [0.84–27.91]	0.088
Early preterm (< 34 wk)	1 (1.9%) [0.3–9.8]	0 (0.0%)	1 (4.8%) [0.8–22.7]	—	0.389
Post term (≥ 41 wk)	7 (13.0%) [6.4–24.3]	6 (18.2%) [8.6–34.4]	1 (4.8%) [0.8–22.7]	0.22 [0.02–2.03]	0.227
Birth weight, grams	3100 (2753–3588)	3120 (2750–3700)	2870 (2140–3350)	MD = −250 g [−640 to + 140]	0.016
Head circumference at birth**	34 (32–35)	34 (33–35)	33 (31–34)	MD = −1.0 cm [−2.2 to + 0.2]	0.125
5-min Apgar score, median	9 (8–9)	9 (8–9)	9 (8–9)	—	0.742
Balloon atrial septostomy^§^	7 (13.0%) [6.4–24.3]	6 (18.2%) [8.6–34.4]	1 (4.8%) [0.8–22.7]	0.22 [0.02–2.03]	0.227
Other interventions before surgery^§^	3 (5.6%) [1.9–15.1]	0 (0.0%)	3 (14.3%) [5.0–34.6]	—	0.054
Age at surgery, days	31 (11–126)	13 (9–127)	57 (30–123)	MD = + 44 days	0.170
Cyanosis before surgery	20 (37.0%) [25.4–50.4]	13 (39.4%) [24.7–56.3]	7 (33.3%) [17.2–54.6]	0.77 [0.24–2.44]	0.654
CPB	47 (87.0%) [75.7–93.6]	31 (93.9%) [80.4–98.3]	16 (76.2%) [54.9–89.4]	0.21 [0.03–1.21]	0.096
30-day mortality	7 (13.0%) [6.4–24.3]	4 (12.1%) [4.8–27.3]	3 (14.3%) [5.0–34.6]	1.21 [0.24–6.08]	1.000
1-year mortality	10 (18.5%) [10.4–30.8]	5 (15.2%) [6.7–30.9]	5 (23.8%) [10.6–45.1]	1.75 [0.43–7.13]	0.486

Continuous data are presented as medians (25th-75th percentile); categorical variables are presented as counts (n, %) with 95% Confidence Intervals [95% CI] and Odds Ratios (OR) with 95% CI as effect size measures for comparisons between non-syndromic and syndromic groups. Continuous variables are compared using Median Difference (MD) with 95% CI. Early term (37–39 weeks GA), Late preterm (34–37 weeks GA), Early preterm (< 34 weeks GA), Post term (≥ 41 weeks GA). *Maternal conditions include hypertension, gestational diabetes and hypo/hyperthyroidism. **Head circumference at birth: 10 measurements missing (6 non-syndromic, 4 syndromic). ^§^Includes catheterization procedures performed before surgery and before preoperative MRI (7 BAS in TGA, 2 PDA stenting, 1 azygos vein stenting)

*BAS* Balloon Atrial Septostomy, *CPB* Cardiopulmonary Bypass, *GA* Gestational Age, *IUGR* Intrauterine Growth Retardation, *MD* Median Difference, *wk* weeks

Cardiac diagnoses of the whole cohort are depicted in Table 2. Most of enrolled patients (*n* = 25, 46%) were affected by conotruncal heart defects (CTHDs), 13 (24%) had a single ventricle physiology (SVP), 9 (17%) presented with a left ventricular outflow tract obstruction (LVOTO), 7 (13%) were affected by other CHD. Among syndromic children, the main diagnosis was SVP (7 children, 33%), while non-syndromic children were mainly affected by CTHDs (19 children, 58%).

**Table 2:** Cardiac diagnoses of the study population

	All Patients (*N* = 54)	Non-Syndromic (*N* = 33)	Syndromic (*N* = 21)	Odds Ratio [95% CI]	*p*-value
Conotruncal Heart Defects	25 (46.3%) [33.7–59.4]	19 (57.6%) [40.8–72.8]	6 (28.6%) [13.8–50.2]	0.30 [0.09–0.96]	0.052
TGA	14 (25.9%) [16.1–38.9]	12 (36.4%) [22.2–53.4]	2 (9.5%) [2.7–28.1]	0.18 [0.04–0.93]	0.054
Simple	13 (24.1%) [14.6–37.0]	11 (33.3%) [19.6–50.3]	2 (9.5%) [2.7–28.1]	0.21 [0.04–1.07]	0.057
Complex	1 (1.9%) [0.3–9.8]	1 (3.0%) [0.5–15.3]	0 (0.0%) [0.0–15.3]	—	1.000
TOF	9 (16.7%) [9.0–28.7]	7 (21.2%) [10.7–37.8]	2 (9.5%) [2.7–28.1]	0.39 [0.07–2.12]	0.456
Classic	6 (11.1%) [5.2–22.2]	4 (12.1%) [4.8–27.3]	2 (9.5%) [2.7–28.1]	0.76 [0.12–4.62]	1.000
TOF-DORV	1 (1.9%) [0.3–9.8]	1 (3.0%) [0.5–15.3]	0 (0.0%) [0.0–15.3]	—	1.000
TOF-pulmonary stenosis/atresia	2 (3.7%) [1.0–12.5]	2 (6.1%) [1.7–19.6]	0 (0.0%) [0.0–15.3]	—	0.518
Truncus Arteriosus	2 (3.7%) [1.0–12.5]	0 (0.0%) [0.0–10.0]	2 (9.5%) [2.7–28.1]	—	0.147
Single Ventricle Physiology	13 (24.1%) [14.6–37.0]	6 (18.2%) [8.6–34.4]	7 (33.3%) [17.2–54.6]	2.25 [0.63–8.01]	0.328
HLHS	3 (5.6%) [1.9–15.1]	3 (9.1%) [3.1–23.6]	0 (0.0%) [0.0–15.3]	—	0.274
AVSD	8 (14.8%) [7.7–26.4]	2 (6.1%) [1.7–19.6]	6 (28.6%) [13.8–50.2]	6.20 [1.11–34.61]	0.045
Tricuspid atresia	2 (3.7%) [1.0–12.5]	1 (3.0%) [0.5–15.3]	1 (4.8%) [0.8–22.7]	1.60 [0.09–27.20]	1.000
Left Ventricular Outflow Tract Obstruction	9 (16.7%) [9.0–28.7]	5 (15.2%) [6.7–30.9]	4 (19.0%) [7.7–38.6]	1.32 [0.31–5.63]	0.722
IAA	5 (9.3%) [4.0–19.9]	2 (6.1%) [1.7–19.6]	3 (14.3%) [5.0–34.6]	2.58 [0.39–17.06]	0.370
CoA	4 (7.4%) [2.9–17.3]	3 (9.1%) [3.1–23.6]	1 (4.8%) [0.8–22.7]	0.50 [0.05–5.20]	0.642
**Other**	7 (13.0%) [6.4–24.3]	3 (9.1%) [3.1–23.6]	4 (19.0%) [7.7–38.6]	2.35 [0.47–11.75]	0.411
Ebstein anomaly	1 (1.9%) [0.3–9.8]	1 (3.0%) [0.5–15.3]	0 (0.0%) [0.0–15.3]	—	1.000
VSD, simple	4 (7.4%) [2.9–17.3]	1 (3.0%) [0.5–15.3]	3 (14.3%) [5.0–34.6]	5.33 [0.51–55.33]	0.287
TAPVR	2 (3.7%) [1.0–12.5]	1 (3.0%) [0.5–15.3]	1 (4.8%) [0.8–22.7]	1.60 [0.09–27.20]	1.000

Data are presented as count (*n*, %) and 95% Confidence Intervals [95% CI] for single proportions. Comparisons between Syndromic and Non-Syndromic patients are reported as Odds Ratios (OR) with corresponding 95% CIs. *AVSD* Atrioventricular Septal Defect, *CoA* Coarctation of the Aorta, *CTHDs* Conotruncal Heart Defects, *HLHS* Hypoplastic Left Heart Syndrome, *IAA* Interrupted Aortic Arch, *LVOTO* Left Ventricular Outflow Tract Obstruction, *SVP* Single Ventricle Physiology, *TAPVR* Total Anomalous Pulmonary Venous Return, *TGA* Transposition of the Great Arteries, *TOF* Tetralogy of Fallot, *VSD* Ventricular Septal Defect

Genetic diagnoses of syndromic children are reported in Table S1. Four patients were diagnosed with Trisomy 21, 2 with DiGeorge syndrome, 2 with heterotaxy syndromes. In addition, 7 patients were suspected of having a syndrome, primarily due to multiple dysmorphisms associated with underlying CHD, but genetic test results were inconclusive or still ongoing.

### Brain MRI findings

Preoperative brain MRI findings are reported in Table 3. Median age at brain MRI was 11 (IQR 6–66) days and surgery was performed 6 (IQR 2–15) days after brain MRI. At least one brain lesion was detected in 24/54 (44%) children, 16/33 non-syndromic and 8/21 syndromic (OR 0.65 [0.21–1.94], *p* = 0.5708). More than one brain lesion was found in 5 (9%). The most frequent lesion was AIS, seen in 11 (20%) children. All these lesions but one had high DWI signal and low ADC values (one patient had two ischemic lesions, one of them with low DWI signal/high ADC values). The main artery involved was the middle cerebral artery. Cerebral sinovenous thrombosis (CSVT) was diagnosed in 5 (9%) children, WMI in 3 (6%), haemorrhages were intraventricular in 2 (4%), intraparenchymal in 2 (4%) and subdural in 6 (11%). In addition, increased subarachnoid spaces were observed in 11/54 (20%) children.

**Table 3:** Preoperative brain MRI findings of the study population

	All patients (*N* = 54)	Non-Syndromic (*N* = 33)	Syndromic (*N* = 21)	OR/MD [95% CI]	*p*-value
Age at MRI, days	11 (6–66)	8 (6–19)	29 (7–70)	MD: + 21.0 [−4.0 to 46.0]	0.1120
Preoperative MRI to surgery, days	6 (2–15)	5 (2–21)	11 (5–33)	MD: + 6.0 [−2.0 to 14.0]	0.0632
Any brain lesion	24 (44%) [31.9–57.8]	16 (48%) [32.5–64.8]	8 (38%) [19.9–59.9]	OR 0.65 [0.21–1.94]	0.5708
1	19 (35%) [23.6–48.7]	13 (39%) [24.7–56.3]	6 (29%) [13.8–50.2]	OR 0.62 [0.19–1.99]	0.5634
2	5 (9%) [4.0–19.9]	3 (9%) [3.1–23.6]	2 (10%) [2.7–28.1]	OR 1.05 [0.16–6.85]	1.0000
≥ 3	0 (0%) [0.0–6.6]	0 (0%) [0.0–10.4]	0 (0%) [0.0–15.5]	—	—
Arterial Ischemic Stroke	11 (20%) [11.8–32.9]	6 (18%) [8.6–34.4]	5 (24%) [10.6–43.8]	OR 1.41 [0.39–5.06]	0.7308
Anterior Choroidal Artery	1 (9%) [0.2–41.3]	1 (17%) [0.4–64.1]	0 (0%) [0.0–52.2]	OR 0 [0.0–18.5]	1.0000
Middle Cerebral Artery	8 (73%) [39.0–94.0]	5 (83%) [35.9–99.6]	3 (60%) [14.7–94.7]	OR 0.30 [0.02–4.85]	0.5455
Posterior Cerebral Artery	2 (18%) [2.3–51.8]	0 (0%) [0.0–45.9]	2 (40%) [5.3–85.3]	OR 8.57 [0.30–243.6]	0.1818
Side left/right/both	3/7/1 (27/64/9%)	2/4/0 (33/67/0%)	1/3/1 (20/60/20%)	—	—
Corticospinal tracts involved	3 (27%) [6.0–61.0]	2 (33%) [4.3–77.7]	1 (20%) [0.5–71.6]	OR 0.50 [0.03–9.10]	1.0000
Basal ganglia or thalamus involved	2 (18%) [2.3–51.8]	1 (17%) [0.4–64.1]	1 (20%) [0.5–71.6]	OR 1.25 [0.05–29.12]	1.0000
Cortex involved	0 (0%) [0.0–28.5]	0 (0%) [0.0–45.9]	0 (0%) [0.0–52.2]	—	—
DWI high/ADC low signal	11 (100%) [71.5–100]	6* (100%) [54.1–100]	5 (100%) [47.8–100]	—	—
Cerebral Sinovenous Thrombosis	5 (9%) [3.1–20.3]	4 (12%) [3.4–28.2]	1 (5%) [0.1–23.8]	OR 0.36 [0.04–3.45]	0.6402
Transverse unilateral	2 (40%) [5.3–85.3]	2 (50%) [6.8–93.2]	0 (0%) [0.0–100]	OR 0.00 [0.00–19.2]	1.0000
Transverse bilateral	1 (20%) [0.5–71.6]	1 (25%) [0.6–80.6]	0 (0%) [0.0–100]	OR 0.00 [0.00–38.4]	1.0000
Transverse and Sigmoid unilateral	2 (40%) [5.3–85.3]	1 (25%) [0.6–80.6]	1 (100%) [2.5–100]	OR 9 [0.18–450.0]	0.4000
White Matter Injury	3 (6%) [1.9–15.1]	3 (9%) [3.1–23.6]	0 (0%) [0.0–15.3]	OR 0.22 [0.01–4.47]	0.2736
Single/Multiple	2/1 (66/33%)	2/1 (66/33%)	0 (0)	—	—
Frontal/Parietal	2/1 (66/33%)	2/1 (66/33%)	0 (0)	—	—
DWI high/ADC low signal	3 (100%) [29.2–100]	3 (100%) [29.2–100]	0 (0)	—	—
Corticospinal tracts involved	0 (0%) [0.0–70.8]	0 (0%) [0.0–70.8]	0 (0)	—	—
Side left/right/both	1/1/1 (33/33/33%)	1/1/1 (33/33/33%)	0 (0)	—	—
Intraventricular Haemorrhage	2 (4%) [1.0–12.5]	0 (0%) [0.0–10.0]	2 (10%) [2.7–28.1]	OR 8.58 [0.39–188.85]	0.1468
Grade III (enlarged ventricles)	1 (50%) [2.5–97.5]	0 (0)	1 (50%) [2.5–97.5]	—	0.3889
Grade IV (periventricular hemorrhagic infarction)	1 (50%) [0.3–9.8]	0 (0)	1 (50%) [2.5–97.5]	—	0.3889
Intraparenchymal Haemorrhage	2 (4%) [1.0–12.5]	1 (3%) [0.5–5.3]	1 (5%) [0.8–22.7]	OR 1.60 [0.09–27.20]	1.0000
Cerebral/Cerebellar/Both	0/1/1 (0/50/50%)	0/1/0 (0/100/0%)	0/0/1 (0/0/100%)	—	—
Subdural Haemorrhage	6 (11%) [5.2–22.2]	5 (15%) [6.7–30.9]	1 (5%) [0.8–22.7]	OR 0.28 [0.03–2.53]	0.3863
Supratentorial/Infratentorial/Both	0/3/3 (0/50/50%)	0/3/2 (0/60/40%)	0/0/1 (0/0/100%)	—	—
Hypoxic-Ischemic Watershed Injury	0 (0%) [0.0–6.6]	0 (0%) [0.0–10.4]	0 (0%) [0.0–15.5]	—	—
Increased subarachnoid spaces	11 (20%) [11.8–32.9]	5 (15%) [6.7–30.9]	6 (29%) [13.8–50.2]	OR 2.24 [0.60–8.38]	0.3045
Postoperative brain MRI performed	11 (20%) [11.8–32.9]	3 (9%) [3.1–23.6]	8 (38%) [19.9–59.9]	OR 6.15 [1.42–26.68]	0.0154

Continuous variables are reported as median (IQR). Categorical variables are reported as count (*n*, %) with 95% confidence intervals [95% CI]. Categorical sub-analyses are expressed as proportions of affected patients within each category. *One patient had two ischemic lesions, one of them with low DWI signal/high ADC values. *ADC* Apparent Diffusion Coefficient, *DWI* Diffusion-Weighted Imaging, *MRI* Magnetic Resonance Imaging

In our univariate analysis, although a higher proportion of infants with preoperative brain injury were born before 37 weeks compared with those without injury (25% vs. 10%), the difference did not reach statistical significance (*p* = 0.2783).

**Fig. 2:**
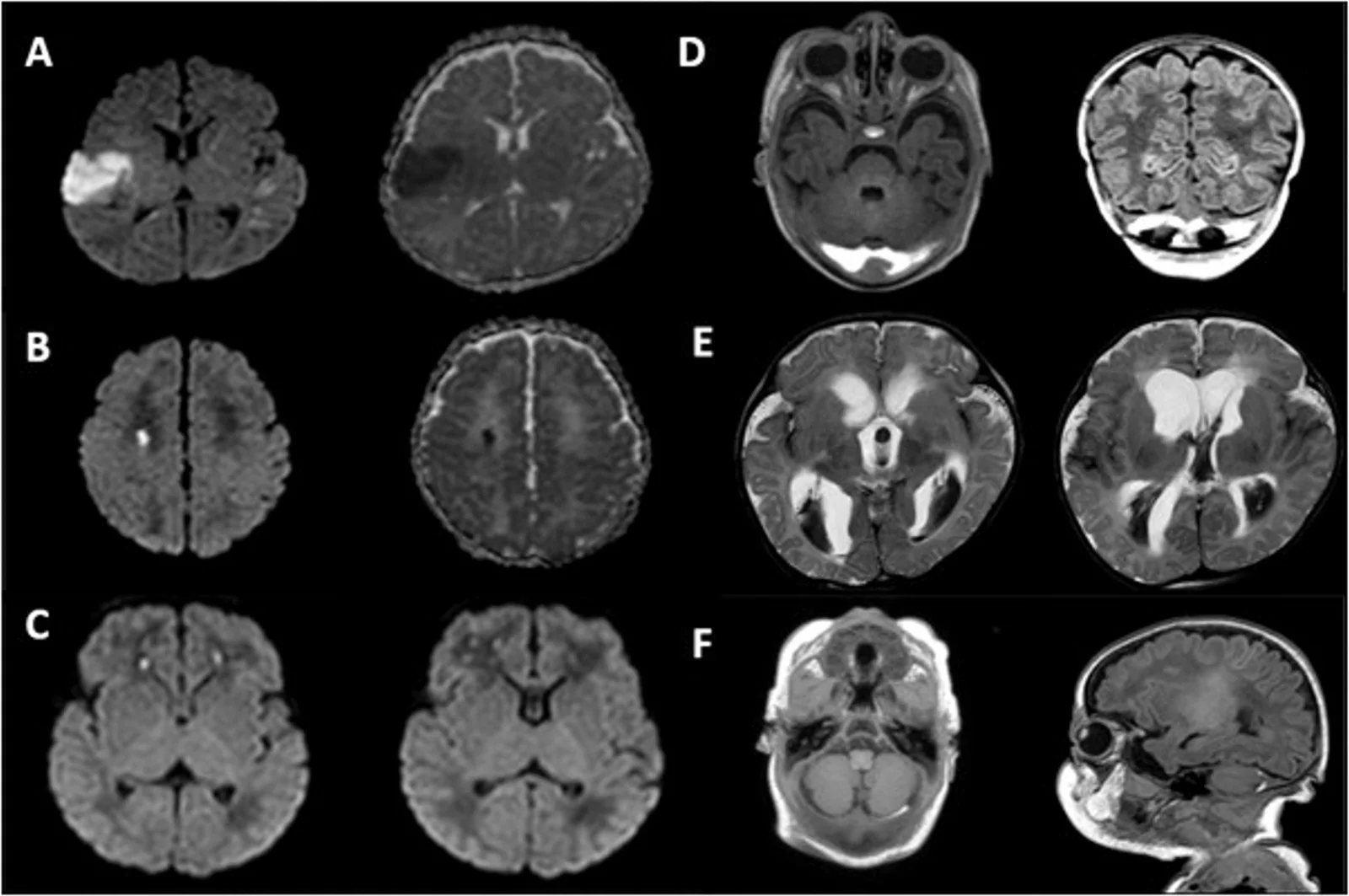
Examples of preoperative brain MRI findings in the study population. **A** Recent ischemic lesion in the right middle cerebral artery territory, with high DWI/ADC low signal (recent injury). **B** Recent ischemic lesion (DWI/ADC) in the right anterior choroidal artery territory. **C** Axial diffusion weighted images showing multiple white matter injuries in the frontal white matter, bilaterally. **D** Axial T1 and coronal FLAIR images showing thrombosis of both transverse sinuses. **E** Axial T2 images showing massive intraventricular haemorrhage (grade III) with hypointense blood clots in the III ventricle and in the atria of the lateral ventricles; the supratentorial ventricular system is markedly enlarged. **F** Axial and sagittal T1 images showing a small hyperintense left infratentorial subdural hematoma. (Courtesy of R.M., Neuroradiology, Department of Neurosciences, University of Padova, Italy)

Examples of preoperative MRI findings in our cohort can be seen in Fig. 2. Potential risk factors and their association with preoperative brain lesions in our cohort are shown in Table 4. No statistically significant association was found.

**Table 4:** Potential risk factors for preoperative brain injury in the study population

	No brain injury (*N* = 30)	Brain injury (*N* = 24)	OR [95% CI]	*p*-value
Maternal conditions	7 (23.3%) [9.9–42.3]	4 (16.7%) [4.7–37.4]	0.66 [0.16–2.65]	0.7363
No prenatal diagnosis	10 (33.3%) [17.3–52.8]	7 (29.2%) [12.6–51.1]	0.82 [0.25–2.68]	0.7768
Male sex	20 (66.7%) [47.2–82.7]	14 (58.3%) [36.6–77.9]	0.70 [0.23–2.14]	0.5798
< 37 weeks GA	3 (10.0%) [2.1–26.5]	6 (25.0%) [9.8–46.7]	3.00 [0.65–13.88]	0.2783
Cesarean delivery	12 (40.0%) [22.7–59.4]	10 (41.7%) [22.1–63.4]	1.07 [0.36–3.20]	1.0000
Vaginal induced delivery	4 (13.3%) [3.8–30.7]	5 (20.8%) [7.1–42.2]	1.71 [0.40–7.28]	0.4893
APGAR ≤ 5 (at 5 min after birth)	4 (13.3%) [3.8–30.7]	2 (8.3%) [1.0–27.0]	0.59 [0.10–3.57]	0.6818
CTHDs	15 (50.0%) [31.3–68.7]	10 (41.7%) [22.1–63.4]	0.71 [0.24–2.10]	0.5919
SVP	8 (26.7%) [12.3–45.9]	5 (20.8%) [7.1–42.2]	0.72 [0.20–2.61]	0.7527
LVOTO	4 (13.3%) [3.8–30.7]	5 (20.8%) [7.1–42.2]	1.71 [0.40–7.28]	0.4893
Cyanosis before surgery	8 (26.7%) [12.3–45.9]	12 (50.0%) [29.1–70.9]	2.75 [0.88–8.56]	0.1471
BAS	3 (10.0%) [2.1–26.5]	4 (16.7%) [4.7–37.4]	1.80 [0.36–8.99]	0.6868
> 30 days old at surgery	17 (56.7%) [37.4–74.5]	10 (41.7%) [22.1–63.4]	0.55 [0.18–1.63]	0.4117
Abnormal preop. neuro exam	18 (60.0%) [40.6–77.3]	11 (45.8%) [25.6–67.2]	0.56 [0.19–1.68]	0.4112

Data are presented as numbers (*n*, %) and 95% CI. *BAS* Balloon Atrial Septostomy, *CTHDs* Conotruncal Heart Defects, *GA* Gestational Age, *LVOTO* Left Ventricular Outflow Tract Obstruction, *SVP* Single Ventricle Physiology

Postoperative brain MRI was performed, on a clinical basis, in 11/54 (20%, 3 non-syndromic and 8 syndromic) children, whose clinical characteristics can be found in Table S2. Global cerebral atrophy was found in 5/11 (46%) children, all of them syndromic, microhaemorrhages in 4/11 (36%), new ischemic lesions in 2/11 (18%).

### Neurological and neuropsychological outcome

Characteristics of non-syndromic and syndromic children who underwent preoperative and follow-up neurological evaluations are reported in Table 5.

**Table 5:** Preoperative and follow-up neurological evaluations in non-syndromic and syndromic children

	All (*N* = 54)	Non-syndromic (*N* = 33)	Syndromic (*N* = 21)	OR [95% CI]	*p*-value
Preoperative neurological evaluation	44 (81.5%) [68.6–90.7]	28 (84.8%) [68.1–94.9]	16 (76.2%) [52.8–91.8]	0.57 [0.15–2.22]	0.4891
Axial hypotonia	24 (54.5%) [38.8–69.6]	12 (42.9%) [24.5–62.8]	12 (75.0%) [47.6–92.7]	4.00 [1.03–15.53]	0.0600
Psychomotor delay	4 (9.1%) [2.5–21.7]	0 (0.0%) [0.0–12.3]	4 (25.0%) [7.3–52.4]	20.52 [1.03–410.70]	0.0134
Preoperative seizures	3 (6.8%) [1.4–18.7]	2 (7.1%) [0.9–23.5]	1 (6.3%) [0.2–30.2]	0.87 [0.07–10.38]	1.0000
Follow-up neurological evaluation	35 (64.8%) [50.6–77.3]	24 (72.7%) [54.5–86.7]	11 (52.4%) [29.8–74.3]	0.41 [0.13–1.31]	0.1472
Language delay	19 (54.3%) [36.6–71.2]	10 (41.7%) [22.1–63.4]	9 (81.8%) [48.2–97.7]	6.30 [1.11–35.67]	0.0354
Motor delay	13 (37.1%) [21.5–55.1]	5 (20.8%) [7.1–42.2]	8 (72.7%) [39.0–94.0]	10.13 [1.94–52.90]	0.0069
Neuropsychiatric symptoms	4 (11.4%) [3.2–26.7]	4 (16.7%) [4.7–37.4]	0 (0.0%) [0.0–28.5]	0.19 [0.01–3.83]	0.2849
Seizures or epilepsy	5 (14.3%) [4.8–30.3]	0 (0.0%) [0.0–14.2]	5 (45.5%) [16.7–76.6]	41.46 [2.02–850.70]	0.0019

Data are presented as count (n, %) and 95% CI. P value was calculated by Fisher’s exact test, and we considered a significant result at *p* < 0.05. OR with 95% CI and *p*-values assess differences between non-syndromic and syndromic cohorts. **We included children who were evaluated at least once during follow-up

Preoperative neurological evaluation was available in 44/54 children (81%; 28 males; 28 non-syndromic and 16 syndromic). Axial hypotonia was diagnosed in 12/28 (42%) non-syndromic and 12/16 (75%) syndromic children (OR 4.00 [1.03–15.53], *p* = 0.0600). Psychomotor delay was evident in 4/16 (25%) syndromic and in 0/33 non-syndromic children (OR 20.52 [1.03–410.70], *p* = 0.0134). Two non-syndromic children suffered from preoperative seizures: one child in neonatal period and his brain MRI showed two ischemic lesions; one child suffered from acute provoked seizures during a preoperative sepsis episode and his brain MRI showed bilateral transverse CSVT. Both did not develop epilepsy at the following neurological evaluations. One syndromic child suffered from preoperative symptomatic seizures due to cardioembolic stroke. One syndromic child diagnosed with tricuspid atresia showed early signs of severe motor impairment (tetraparesis) on preoperative neurological evaluation, and his brain MRI showed both grade III IVH and CSVT. Genetic testing was inconclusive.

At least one follow-up neurological evaluation was available for 35/54 children (65%; 24 were non-syndromic and 11 syndromic, 31/35 underwent preoperative evaluation).

Ten (42%) non-syndromic and nine (82%) syndromic children showed a language delay (OR 6.30 [1.11–35.67], *p* = 0.0354). Five (20%) non-syndromic and eight (73%) syndromic showed a motor delay (OR 10.13 [1.94–52.90], *p* = 0.0069). Four (17%) non-syndromic children showed neuropsychiatric symptoms (e.g. hyperactivity). Five (45%) syndromic children suffered from postoperative seizures: two were diagnosed with focal symptomatic epilepsy after a postoperative ischemic stroke, one has a developmental and epileptic encephalopathy (SALL4 gene mutation) and one child had only one isolated seizure in the postoperative period.

Stratified Mantel–Haenszel analyses confirmed that syndromic status remained significantly associated with both language delay and motor delay at follow-up after adjusting for baseline confounders (gestational age < 37 weeks, IUGR, birth weight < 2500 g, caesarean delivery).

A sensitivity analysis was performed excluding the 7 patients with unconfirmed genetic syndrome (*n* = 14 confirmed syndromic vs. *n* = 33 non-syndromic). The analysis yielded consistent results: the prevalence of preoperative brain MRI lesions remained comparable between groups (36% vs 48%; OR 0.59 [0.16–2.18], *p* = 0.528), whereas confirmed syndromic children maintained significantly higher rates of motor delay (75% vs 20%; OR 12.00 [1.83–78.68], *p* = 0.009) and language delay (88% vs 42%; OR 9.33 [1.02–85.71], *p* = 0.041).

No statistically significant association was observed between the presence of preoperative brain MRI lesions and subsequent motor or language delay. Specifically, motor and/or language delay was identified in 7 out of 14 patients (50.0%) with preoperative brain injury compared to 13 out of 21 patients (61.9%) with normal preoperative MRI scans (OR = 0.62, [0.16–2.42], *p* = 0.501). Subgroup analysis stratified by genetic status further confirmed this lack of association.

Regarding missing data, Little's MCAR test was performed across all clinical variables with missing values (birth head circumference, preoperative neurological evaluation, postoperative MRI and follow-up neurological assessment). The test was consistent with data being missing completely at random (χ2 = 14.82, df = 11, *p* = 0.191), suggesting that missingness was completely random and is unlikely to have introduced systematic bias.

## Discussion

### Main findings: syndromic vs non-syndromic cohort

To the best of our knowledge, this is the first study to compare preoperative brain lesions and neurodevelopmental outcomes between syndromic and non-syndromic children affected by CHD.

Most existing literature has focused on non-syndromic patients, primarily to minimise confounding factors related to underlying genetic conditions. However, we believe that including syndromic children is of significant clinical relevance, particularly because clinical and surgical decision-making often occurs before genetic test results become available.

Preoperative brain injury is an established independent predictor of adverse neurodevelopmental outcomes, reflecting cerebral vulnerability prior to cardiac surgery. In our cohort, no significant differences were observed between syndromic and non-syndromic patients in the number, type or severity of preoperative brain lesions, including white matter injury, arterial ischemic stroke, haemorrhage and cerebral sinovenous thrombosis. Only one prior study included patients with known or suspected chromosomal disorders and reported minimal association between chromosomal abnormalities and brain MRI findings [14].

Nevertheless, our subcohort of syndromic children experienced a more complex perinatal course characterized by lower birth weights, higher rates of IUGR and caesarean delivery. In addition, their clinical spectrum was further compounded by a higher severity of cardiac diagnoses. Notably, a high prevalence of twin pregnancies was observed in our cohort, especially among the syndromic children. While the increased risk of CHD in multiple gestations has been extensively described, to our knowledge no study had previously reported the prevalence of twin pregnancies among syndromic children with CHD [15]. The overall higher rate of caesarean sections in our cohort of syndromic infants can likely be attributed to the elevated prevalence of prenatal risk factors or to physician/hospital policy for elective surgical delivery in the presence of recognized dysmorphisms, extracardiac anomalies, or severe complex heart lesions on prenatal screening. Moreover, compared to non-syndromic peers, syndromic children showed higher rates of axial hypotonia and psychomotor delay on preoperative neurological evaluations, indicating a global impairment independent of macrostructural MRI lesions. Besides, preoperative axial hypotonia likely reflects a combination of prolonged hospital admission, bedridden status, and reduced environmental stimulation typical of infants awaiting cardiac surgery, alongside intrinsic genetic vulnerabilities in the syndromic subgroup.

Moreover, during post-surgical follow-up, the syndromic subgroup continued to display higher rates of language delay, motor delay and seizures or epilepsy compared to non-syndromic peers with comparable rates of preoperative brain lesions. While neurodevelopmental outcomes in CHD children are inherently multifactorial, reflecting foetal, perinatal, perioperative and surgical variables, this disparity highlights an underlying vulnerability in the syndromic cohort.

Furthermore, we found no direct correlation between preoperative brain lesions and poor neurodevelopmental outcomes, in either the syndromic or non-syndromic subcohort. This result further confirms that neurodevelopmental outcome is multifactorial and may be driven by microstructural or functional network alterations rather than overt macrostructural brain damage, as supported by recent literature [16].

### Preoperative brain lesions and risk factors

When considering the whole cohort, preoperative brain MRI revealed at least one structural lesion (WMI, ischemia, haemorrhage, CSVT) in 44% of all children. This prevalence aligns with previously published data, particularly considering that subdural hematomas were included in the total count [8, 10–13]. The most frequent lesion in our cohort was AIS, observed in 20% of all children, consistent with previously reported data [11, 17, 18]. The main affected artery was the middle cerebral artery, mirroring findings from the European Association Brain in Congenital Heart Disease Consortium, which reported prevalence of middle cerebral artery involvement in preoperative AIS, in contrast to postoperative strokes, which predominantly affect basal ganglia/thalamus region [12]. CSVT occurred in 9% of our cohort and the transverse sinus was most commonly affected, as previously reported [11]. Conversely, in our cohort the prevalence of WMI in preoperative MRI was 6%, low if compared to extensively reported data [11, 12].

Although not statistically significant, a higher prevalence of enlarged subarachnoid spaces was observed in the syndromic population, consistent with previous reports in children with genetic disorders [19, 20].

Several risk factors for preoperative brain injury detectable on MRI have been identified in the literature, including gestational age, 5-min APGAR score, preoperative cyanosis, specific heart defects (CoA, SVP), BAS, low cardiac output syndrome, altered maternal–fetal environment, male sex, older age at surgery and induced vaginal delivery [11, 13, 14, 21]. In the present study, we explored these variables alongside other potential risk factors. However, no statistically significant association was observed, probably due to low sample size. Nonetheless, a trend towards lower gestational age and lower preoperative peripheral oxygen saturation in children with preoperative brain injury was noted.

### Limitations

This study has several limitations that warrant consideration. First, it was conducted at a single tertiary centre, which is a referral institution for patients with complex CHD, therefore findings may not be generalizable to the broader CHD population. The modest sample size restricted our ability to perform subgroup analyses and limited statistical power for certain secondary endpoints.

Furthermore, a potential selection bias exists, as critically ill infants with severe hemodynamic instability or life-threatening conditions were systematically excluded from our analyses. These critically ill infants are potentially at the highest risk for both pre- and postoperative brain injury and adverse neurodevelopmental outcomes. Consequently, our findings should be interpreted with caution when generalized to the broader CHD population.

Unconventionally, premature infants were included in our sample. Although preterm birth is an established independent risk factor for both brain injury and adverse neurodevelopmental outcomes, prematurity was not associated with a higher risk of these conditions in our univariate analyses. Nonetheless, it remains a potential confounder and our findings should be interpreted with caution.

Moreover, we acknowledge methodological limitations regarding the classification of the syndromic group. Diagnostic misclassification could have occurred considering that 7/21 syndromic patients had a suspected, rather than genetically confirmed, syndrome. However, a sensitivity analysis excluding unconfirmed cases yielded consistent results: the prevalence of preoperative brain MRI lesions remained comparable between groups, while confirmed syndromic children maintained significantly higher rates of motor and language delays. Additionally, substantial genetic heterogeneity was present within the syndromic group, encompassing conditions with distinct neurodevelopmental trajectories and disease-specific risks for cognitive, motor, and language impairments. While grouping these different genetic conditions was necessary due to sample size considerations, future multicentre studies with larger cohorts are needed to isolate the relative contributions of specific genetic aetiologies versus structural brain injury on long-term neurodevelopmental outcomes.

Regarding neurodevelopmental assessments, neurologic evaluations were conducted using standardized scales, such as Hammersmith Neonatal and Infant Neurological Examination scales, as a reference, but only qualitative data were collected [22]. Implementing quantitative developmental scales preoperatively and during follow up would provide a more precise stratification of neurodevelopmental risk and track multi-domain developmental progress more objectively [23, 24]. Another limitation in assessing the language domain was that the study did not account for whether the participants' families were monolingual or bilingual.

Regarding brain MRI examinations, the study lacks quantitative brain MRI metrics, which have been utilized in recent literature to detect subtle structural alterations [16]. A limited number of postoperative brain MRI scans were performed, as post-surgical neuroimaging was obtained solely for clinical indications rather than systematically.

Regarding statistical limitations, univariate analyses were not corrected for multiple testing; thus, secondary findings must be interpreted as exploratory. Furthermore, statistical power limitations in this modest cohort precluded multivariable adjusted models, meaning that residual confounding cannot be entirely ruled out, and our results should be validated in larger prospective multicentre cohorts.

## Conclusions

Neurodevelopmental outcome is one of the main concerns in children with CHD. Preoperative brain lesions reflect foetal and perinatal brain growth and susceptibility to damage and represent an independent risk factor for neurodevelopmental outcome.

We found a high prevalence of preoperative brain injuries in our cohort of children with CHD, mainly ischemic lesions, as already reported in literature. The key and novel finding of our study is that syndromic children with CHD do not show increased rates of preoperative brain injury compared to non-syndromic children, although they remain a population at higher neurodevelopmental risk. We believe that the present study adds new insights on preoperative risk factors for neurodevelopmental outcome in children with CHD, especially in those with genetic syndromes, that have been so far excluded from most of the literature. Further studies are needed to validate our results.

## Supplementary Information

Below is the link to the electronic supplementary material.

Supplementary file1 (DOCX 21 KB)

## Data Availability

The datasets generated during and/or analyzed during the current study are available from the corresponding author on reasonable request.

## Abbreviations

AIS: Arterial Ischemic Stroke
CHD: Congenital Heart Disease
CPB: Cardiopulmonary Bypass
CSVT: Cerebral Sinovenous Thrombosis
DWI: Diffusion-Weighted Imaging
IVH: Intraventricular Haemorrhage
MRI: Magnetic Resonance Imaging
SDH: Subdural Haemorrhage
SWI: Susceptibility-Weighted Imaging
TGA: Transposition of the Great Arteries
TOF: Tetralogy of Fallot
