# Supplementary material for: Preoperative brain injury and neurodevelopmental outcomes in syndromic and non-syndromic children with complex congenital heart disease: a single-centre cohort study
Source: Eur J Pediatr. 2026 Sep 29;185(10):782. doi: 10.1007/s00431-026-07437-w (PMC13623767; doi:10.1007/s00431-026-07437-w)
Supplement: Supplementary file 1 [file 431_2026_7437_MOESM1_ESM.docx]

|  | **Syndromic children**  **N=21** |
| --- | --- |
| **Trisomy 21** | 4 |
| **Di George** | 2 |
| **Heterotaxy** | 2 |
| **CHARGE** | 1 |
| **PHACES** | 1 |
| **10q26 deletion** | 1 |
| **Schimmelpenning Feuerstein-Mims** | 1 |
| **SALL4 gene mutation** | 1 |
| **VACTERL** | 1 |
| **Suspected syndrome*** | 7 |

***Table S1***: Genetic syndromes (diagnosed or suspected) in syndromic children (N=21).

*Suspected syndrome: children with multiple dysmorphisms, inconclusive or ongoing genetic testing.

CHARGE: Coloboma, Heart Defects, Atresia of the Choanae, Retardation of Growth/Development, Genital Anomalies, Ear Abnormalities; PHACES: Posterior fossa malformations, Hemangioma, Arterial anomalies, Cardiac anomalies, Eye abnormalities, Sternal clefting/supraumbilical raphe; VACTERL: Vertebral defects, Anal atresia, Cardiac defects, Tracheo-Esophageal fistula/atresia, Renal anomalies, Limb abnormalities.

|  | **Genetic syndrome** | **Cardiac diagnosis** | **Preoperative MR** | **Surgery to postoperative MR, days** | **New brain lesions**  **on postoperative MR** |
| --- | --- | --- | --- | --- | --- |
| #1 | no | TOF-DORV | increased subarachnoid spaces | 385 | cerebral and cerebellar microhemorrhages |
| #2 | no | simple TGA | ischemic lesion | 8 | IVH grade III |
| #3 | no | Ebstein | CSVT | 30 | recent ischemic lesion |
| #4 | CHARGE | simple TGA | increased subarachnoid spaces | 46 | cerebral microhemorrhages,  global cerebral atrophy |
| #5 | PHACES | IAA | cerebellar dysplasia | 204 | - |
| #6 | 10q26 deletion syndrome | Truncus arteriosus | normal | 21 | SDH, global cerebral atrophy, dilated cerebral ventricles |
| #7 | suspected | TOF | normal | 17 | global cerebral atrophy |
| #8 | suspected | IAA | normal | 47 | microhemorrhages,  global cerebral atrophy |
| #9 | VACTERL | VSD | IVH grade IV | 32 | SDH |
| #10 | suspected | Tricuspid atresia | IVH grade III, CSVT | 8 | - |
| #11 | Okihiro syndrome | AVSD | ischemic lesion | 567 | ischemic lesion, cerebral microhemorrhages, global cerebral atrophy |

***Table S2***: New postoperative brain lesions and characteristics of children who underwent both preoperative and postoperative brain MR (N=11, 3 non-syndromic and 8 syndromic). All of them underwent cardiac surgery with CPB. AVSD: Atrioventricular Septal Defect; CHARGE: Coloboma, Heart Defects, Atresia of the Choanae, Retardation of Growth/Development, Genital Anomalies, Ear Abnormalities; CSVT: Cerebral Sinovenous Thrombosis; IAA: Interrupted Aortic Arch; IVH: Intraventricular Hemorrhage; PHACES: Posterior fossa malformations, Hemangioma, Arterial anomalies, Cardiac anomalies, Eye abnormalities, Sternal clefting/supraumbilical raphe; SDH: Subdural Hemorrhage; TGA: Transposition of Great Arteries; TOF: Tetralogy of Fallot; TOF-DORV: Tetralogy of Fallot-Double Outlet Right Ventricle; VACTERL: Vertebral defects, Anal atresia, Cardiac defects, Tracheo-Esophageal fistula/atresia, Renal anomalies, Limb abnormalities; VSD: Ventricular Septal Defect.
